# Free-running Sn precipitates: an efficient phase separation mechanism for metastable Ge_1−x_Sn_x_ epilayers

**DOI:** 10.1038/s41598-017-16356-8

**Published:** 2017-11-23

**Authors:** Heiko Groiss, Martin Glaser, Magdalena Schatzl, Moritz Brehm, Dagmar Gerthsen, Dietmar Roth, Peter Bauer, Friedrich Schäffler

**Affiliations:** 1 Institute of Semiconductor and Solid State Physics, Johannes Kepler University Linz, Altenberger Str. 69, 4040 Linz, Austria; 2 Center of Surface and Nanoanalytics (ZONA), Johannes Kepler University Linz, Altenberger Str. 69, 4040 Linz, Austria; 3 CEST Competence Center for Electrochemical Surface Technology, Viktor Kaplan Straße 2, 2700 Wiener Neustadt, Austria; 4 Laboratory for Electron Microscopy, Karlsruhe Institute of Technology, Engesserstr. 7, 76131 Karlsruhe, Germany; 5 Institute of Experimental Physics, Division Atomic Physics and Surface Science, Johannes Kepler University Linz, Altenberger Str. 69, 4040 Linz, Austria

## Abstract

The revival of interest in Ge_1−x_Sn_x_ alloys with x ≥ 10% is mainly owed to the recent demonstration of optical gain in this group-IV heterosystem. Yet, Ge and Sn are immiscible over about 98% of the composition range, which renders epilayers based on this material system inherently metastable. Here, we address the temperature stability of pseudomorphic Ge_1−x_Sn_x_ films grown by molecular beam epitaxy. Both the growth temperature dependence and the influence of post-growth annealing steps were investigated. In either case we observe that the decomposition of epilayers with Sn concentrations of around 10% sets in above ≈230 °C, the eutectic temperature of the Ge/Sn system. Time-resolved *in-situ* annealing experiments in a scanning electron microscope reveal the crucial role of liquid Sn precipitates in this phase separation process. Driven by a gradient of the chemical potential, the Sn droplets move on the surface along preferential crystallographic directions, thereby taking up Sn and Ge from the strained Ge_1−x_Sn_x_ layer. While Sn-uptake increases the volume of the melt, single-crystalline Ge becomes re-deposited by a liquid-phase epitaxial process at the trailing edge of the droplet. This process makes phase separation of metastable GeSn layers particularly efficient at rather low temperatures.

## Introduction

In the last few years, interest in direct-gap group-IV heterostructures^1–4^ has mainly been driven by the search for CMOS-compatible light emitters for monolithically integrated optical communication devices^5,6^, or for on-chip optical interconnects^7–9^. Sn-containing group-IV alloys are attractive because their band gap can be engineered all the way from a semiconductor to a semimetal^10^, and, moreover, they are the only known group-IV semiconductors that can assume a direct band gap without additional strain- or defect engineering^11–14^. In this respect, a seminal breakthrough was achieved with the recent demonstration of lasing in strain-relaxed Ge_1−x_Sn_x_ epilayers with Sn concentrations around 10%^15,16^.

Despite this major accomplishment, the thermal stability of Ge_1−x_Sn_x_ films with such high Sn concentrations has always been a concern. Sn has a lattice mismatch of 14.6% with respect to Ge concomitant with a miscibility gap over almost the entire composition range^17^. Moreover, at 13.2 °C pure Sn undergoes an allotropic phase transition from the diamond lattice of semi-metallic α-Sn to the tetragonal lattice of metallic β-Sn. Thus, a precondition for utilizing Ge_1−x_Sn_x_ alloys in meaningful device applications is the feasibility of metastable epitaxial growth with Sn concentrations far above the solid solubility limit of x_sl_ ≈ 1%^17^.

Over the last 30 years a wide range of growth parameters has been investigated aiming at the implementation of metastable epitaxial films and layer sequences containing Si_1-y_Sn_y_ or Ge_1−x_Sn_x_ alloys. It has been demonstrated that far from thermal equilibrium metastable Ge_1−x_Sn_x_ epilayers can be grown with x ≫ x_sl_^11,15,18–24^. Still, for CMOS-compatible device integration the low eutectic temperature T_EC_ = 231 °C of the binary Ge-Sn alloy, which is less than 1 °C lower than the melting point of Sn^17^, remains a problem.

The temperature stability of substitutionally incorporated Sn in diamond-type host lattices has been investigated by several groups^11,22,23,25–29^. In particular, Sn precipitation at the growth front of Ge_1−x_Sn_x_ films on Ge(001) was observed already at growth temperatures T_G_ > 150 °C^21,30^. Also, the authors of ref.^30^ were the first to report trails behind the Sn precipitates as evidence for their movement over the surface. Moving Sn precipitates have meanwhile been confirmed by several groups on material either grown by molecular beam epitaxy (MBE)^31–33^ or by the CVD (chemical vapor deposition) process^34^ that led to the first demonstration of lasing in this material system^15^.

Still, little is known about the mechanisms that propel Sn precipitates while simultaneously, as we will demonstrate in this contribution, inducing the phase separation of the underlying GeSn films into its constituents Ge and Sn. Such a mechanism can jeopardize any application of the GeSn heterosystem that requires processing and/or operation temperatures above T_EC_. Therefore, a comprehensive understanding of the underlying physics is required to devise measures that can suppress this type of phase separation at comparably low temperatures in device applications.

For this purpose, we conducted systematic growth and annealing experiments on uncapped Ge_1−x_Sn_x_ films grown by MBE on Ge(001). Commercial Ge(001) substrates were employed to rule out any influence of the high threading dislocation densities and local strain variations^35^ typically associated with Ge-buffered Si substrates, alias *virtual substrates* ^28,36^. Details of layer growth and the subsequent characterization by X-ray diffraction (XRD), Rutherford back-scattering (RBS), scanning electron microscopy (SEM), atomic force microscopy (AFM), transmission electron microscopy (TEM) and energy dispersive X-ray spectroscopy (EDXS) are described in the section on *experimental methods*. The growth parameters of the four sample series A–D used in this study are listed in Table 1, and experimental details complementing the results in the main text are given in sections S1–S4 in the Supplementary Materials.

**Table 1:** Growth and annealing parameters of the investigated samples.

sample#	T_G_ (°C)	T_A_ (°C)	x_RBS_ (%)	x_XRD_ (%)	d (nm)	r (nm/s)
**Series A**						
A1	120	—	5.13	4.9	30	0.015
A2	120	—	6.70	7.3	30	0.015
A3	120	—	9.25	9.4	30	0.015
A4	120	—	12.70	12.8	30	0.015
A5	120	—	—	13.6	30	0.015
A6	120	—	—	14.5	30	0.015
A7	120	—	—	15.0	30	0.015
A8	120	—	—	5.0	100	0.015
A9	200		11.00	11.5	100	0.1
A10	200	—	—	8.2	250	0.1
**Series B**						
B1	150	—	—	10.1	50	0.1
B2	200	—	—	10.1	50	0.1
B3	225	—	—	—	50	0.1
B4	250	—	—	—	50	0.1
B5	275	—	—	—	50	0.1
B6	300	—	—	—	50	0.1
**Series C**						
C1 = B2	200	—	—	10.1	50	0.1
C2	200	200	—	10.1	50	0.1
C3	200	275	—	—	50	0.1
C4	200	300	—	—	50	0.1
C5	200	350	—	—	50	0.1
**Series D**						
D1	200	≥230		10.1	50	0.1
D2	200	≥230		10.1	50	0.1

T_G_: growth temperature; T_A_: annealing temperature for a 15 min *in-situ* annealing step in Series C, and *ex-situ*, real time annealing in Series D, respectively; x_RBS_: Sn concentration determined by Rutherford backscattering (RBS); x_XRD_: Sn concentration determined by X-ray diffraction (XRD) assuming Vegard’s law; d: thickness of the Ge_1−x_Sn_x_ film; r: deposition rate.

## Experiments

**Figure 1:**
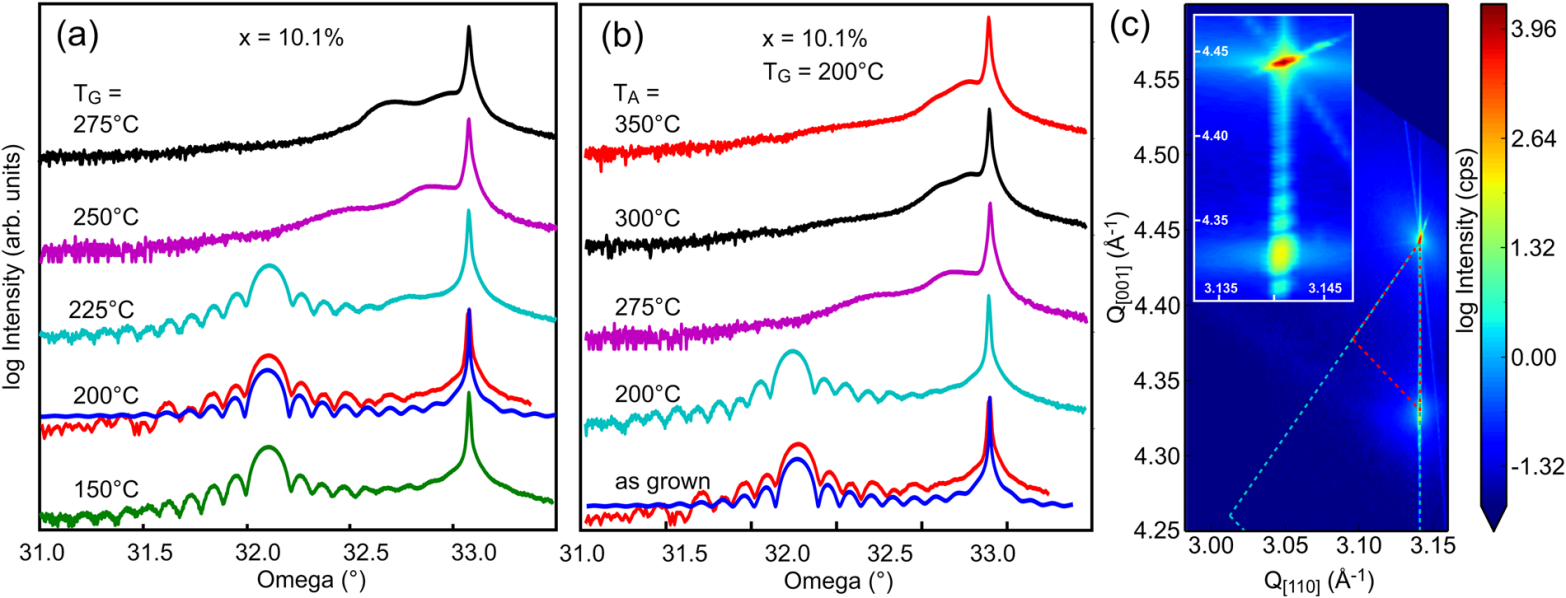
(**a**) X-ray rocking curves from samples of Series B, which were grown at increasing growth temperature T_G._ (**b**) Rocking curves from samples of Series C, which were annealed *in-situ* at increasing annealing temperatures T_A_. (**c**) The 224 reciprocal space map of sample C2 with 10% Sn (turquoise rocking curve in (**b**)) and the magnified inset reveal fully strained (pseudomorphic) epitaxial growth. The curve pairs labeled “200 °C” in (**a**) and “as grown” in (**b**) represent the same sample (B2 = C1 in Table 1), with the respective red line depicting the experimental results, and the blue line representing pendellösung simulations.

Sample series A was grown as a reference for an assessment of the accessible growth conditions in our MBE system. In brief, 30 nm thick Ge_1−x_Sn_x_ films were grown with increasing compositions x either at T_G_ = 120 °C with a deposition rate of r = 0.015 nm/s, or at T_G_ = 200 °C and r = 0.1 nm/s (Table 1). The corresponding X-ray rocking curves show very well-behaved pendellösung fringes^37^ up to x = 14%. As expected^38^, these films are pseudomorphic, i.e. they are free of dislocations and therefore fully strained (Fig. 1(c)). In this composition range, RBS experiments reveal the validity of Vegard's law, i.e. the out-of-plane lattice constant increases linearly with composition x (Fig. S1.1 in the Supplementary Materials). This finding confirms the results in ref.^22^, but disagrees with experiments reported in ref.^23^, where deviations from Vegard's were claimed to set in above x = 8%. TEM investigations did not show extended defects or alloy inhomogeneities up to x = 14%. With higher Sn concentrations, however, the crystal quality decreases rapidly (Fig. S1.2 in the Supplementary Materials), concomitant with the loss of pendellösung fringes in the XRD experiments.

In the following, we concentrate on systematic variations of the growth and annealing temperatures on samples with an application-relevant Sn concentration of x = 10%. Figure 1(a) shows X-ray rocking curves from samples of series B, for which T_G_ was varied between 150 °C and 275 °C (Table 1). Up to T_G_ = 225 °C, i.e. just below T_EC_, the pendellösung fringes are very well resolved, whereas the rocking curves of the two samples grown at 250 °C and 275 °C show only two weak shoulders on the compressively strained side to the Ge 004 substrate peak. Evidently, the homogeneity of the GeSn layers gets lost during film growth above T_EC_.

To further assess this finding, we recorded AFM and SEM images of the degraded samples of series B. These samples show a rough surface with partly embedded droplet-shaped objects. Investigations of sample B6 by TEM (Fig. S2 in the Supplementary Materials) and EDXS revealed that the film between the solidified droplets is single crystalline Ge with a small fraction of dissolved Sn, whereas the droplets consist of β-Sn. Thus, above T_EC_ the Ge_0.9_Sn_0.1_ films become phase-separated already during MBE growth, with the Sn phase segregating at the film surface in liquid form, as inferred from the shape of the precipitates.

**Figure 2:**
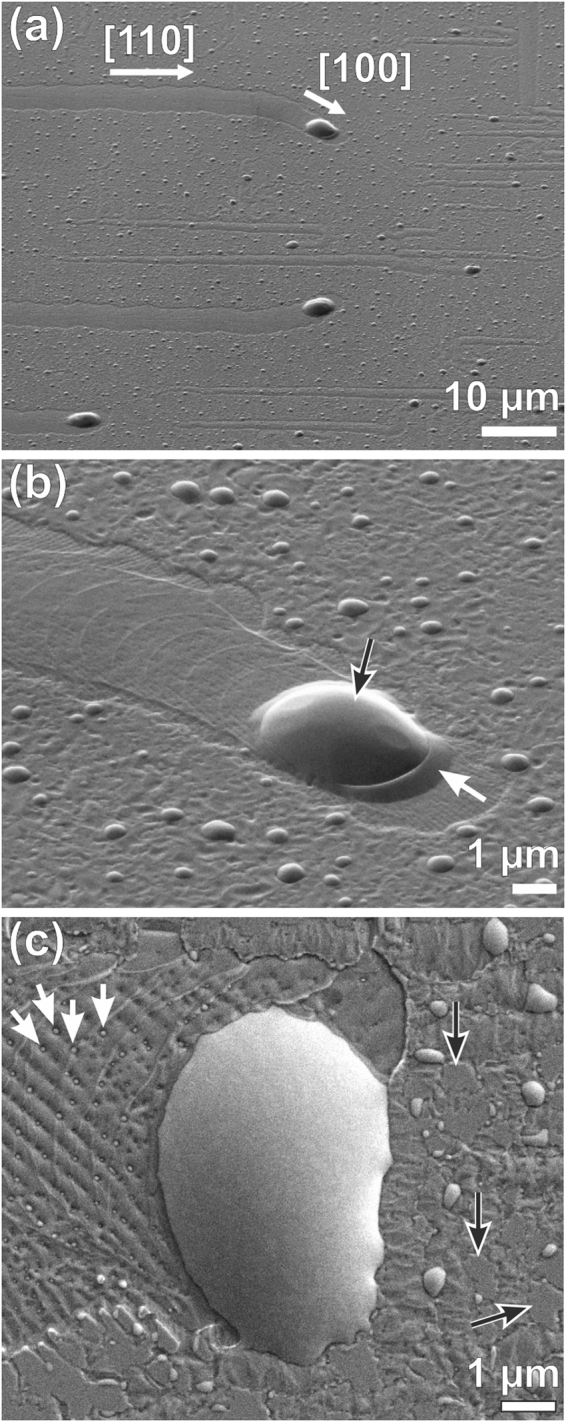
(**a**) and (**b**) SEM overviews of sample C4 after *in-situ* annealing. The trajectories of the Sn droplets follow either <110> or <100> directions. In (**b**) the black arrow marks a solidified, large Sn droplet which is surrounded by a Ge-collar (white arrow). (**c**) Magnified SEM image of a large Sn droplet after *in-situ* annealing. The black arrows mark residual areas of the pristine Ge_0.9_Sn_0.1_ layer. The white arrows point at some of the many tiny Sn droplets that decorate the trail. All images were recorded in the solidified state after cool-down to room temperature.

**Figure 3:**
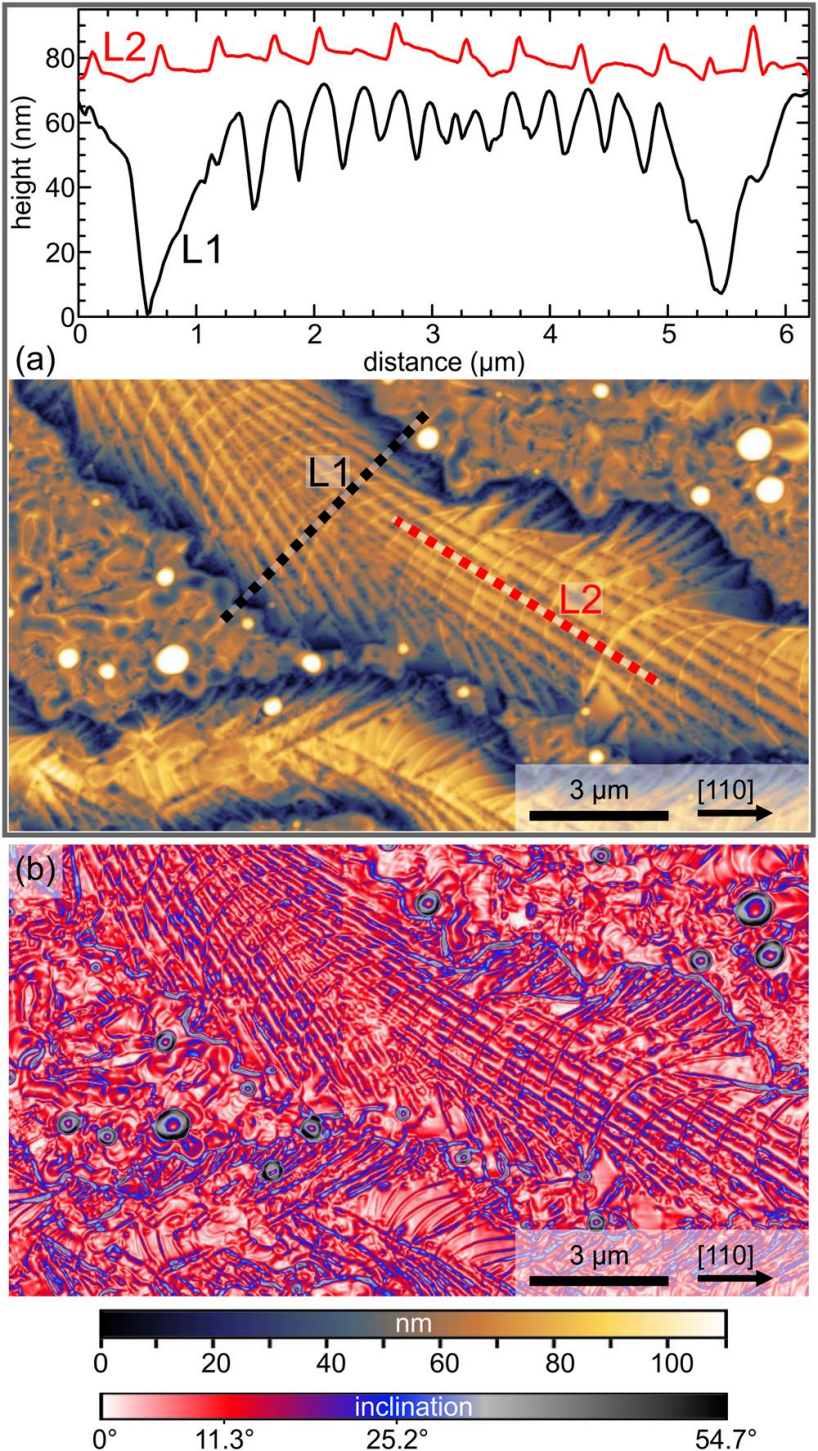
AFM images of sample C4 which was *in-situ* annealed at 300 °C. (**a**) Height image showing a wide Ge trail with its characteristic herring-bone pattern and the distinctive trenches that terminate the trail laterally. The color-coded line scans L1 and L2 shows profiles across and along the upper trail at the locations indicated. 60 nm deep trenches separate the trail region from the adjacent regions that are not affected by the movement of the large Sn droplet. (**b**) Same image displayed as a surface-angle plot indicating the local inclinations with respect to the (001) substrate surface. The color scale is chosen in a way that highlights {001} (0° inclination), {105} (11.3° inclination) and {113} (25.2° inclination) facets, which are well-known low-energy facets of Ge.

A similar temperature dependence was observed on the samples of series C, for which fully strained Ge_0.9_Sn_0.1_ films (Fig. 1(c)) were grown at T_G_ = 200 °C, i.e. below T_EC_ (Table 1). The films were then annealed *in-situ* for 15 min at temperatures T_A_ between 200 °C and 350 °C. As expected, the films remained stable at T_A_ = 200 °C, but higher annealing temperatures led to a complete loss of the pendellösung fringes and the appearance of two weak shoulders at the compressively strained (left) side of the substrate peak (Fig. 1(b)). Figure 2 depicts SEM images after cool-down to room-temperature which reveal the morphological changes that occurred during annealing above T_EC_. Again, droplet-shaped precipitates of varying diameters appear on the surfaces. Distinct trails attached to the largest droplets are predominantly oriented along the <110> directions of the substrate, but trails in the <100> directions are also observed, as well as transitions from one preferred direction class to the other (Fig. 2(a)). SEM images with higher magnification (Fig. 2(b) and (c)), and AFM images (Fig. 3) show a complex fine structure on the trails. The most prominent features are bundles of herringbone-like lines (Fig. 2(c) and Fig. 3) that are speckled with small Sn precipitates (white arrows in Fig. 2(c)). The AFM line scans in Fig. 3(a) reveal that the trails have essentially the same thickness as the original epilayer, except for two ≈ 60 nm deep trenches that confine them laterally. Maps of the local inclination angles (Fig. 3(b)) reveal that the herringbone pattern contains well-known low-energy facets of Ge, namely {001}, {105} and {113}^39^. Between the larger Sn droplets and their trails also smaller precipitates are present which come with their own, more unsteady trails. Only small patches of the surface appear un-affected by droplet movement and phase separation and therefore preserve the smoothness of the original layer surface (Fig. 2(c), black arrows).

**Figure 4:**
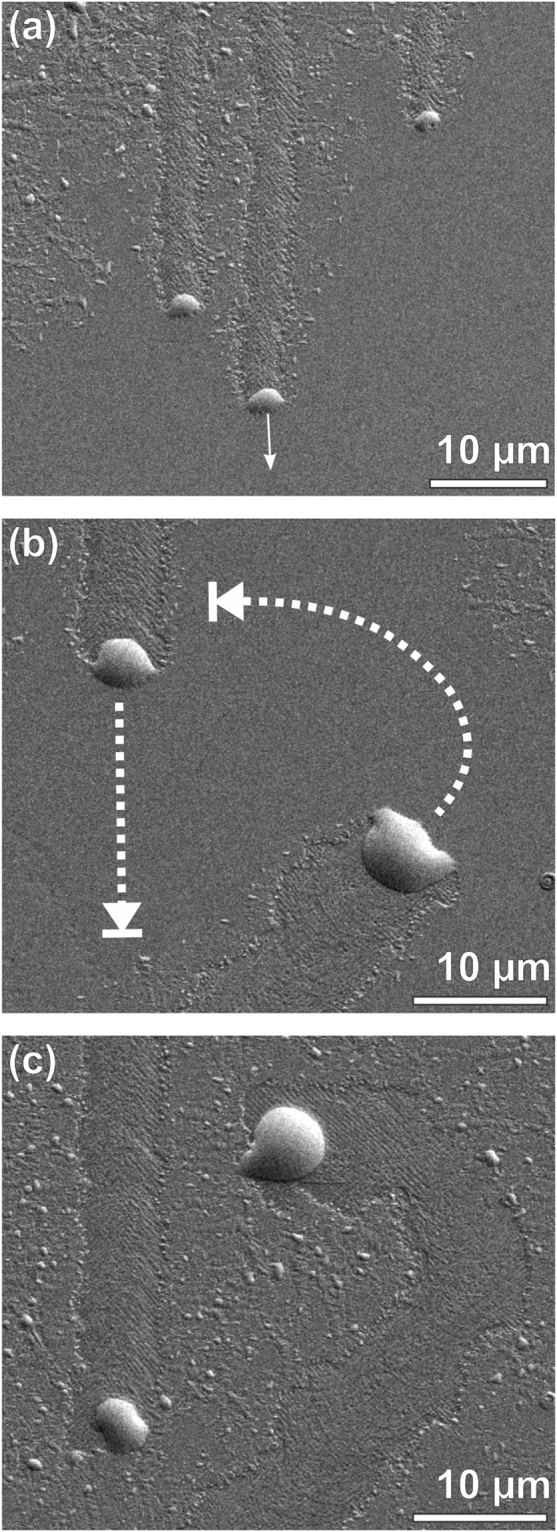
Sequence of SEM images taken from video sequence V1 in the Supplementary Materials. (**a**) Shows the parallel movement of three large Sn droplets along a <110> trajectory (arrow). Image (**b**) depicts the situation shortly before the center droplet in (**a**) intersects the trajectory of another droplet that comes from below on a <100> trajectory. Dotted lines indicate the projected trajectories of the two dots, which both come to a halt once they are completely surrounded by trails of other droplets. The final state with arrested droplet movement is depicted in (**c**). The SEM images were recorded *in-situ*, i.e., the recorded Sn droplets are in the liquid state.

To assess the morphological transformation mechanism in more detail, we performed time-resolved post-growth annealing experiments in the high-vacuum environment of a SEM instrument equipped with a temperature-controlled sample holder. For this purpose, samples from series D were transferred from the MBE chamber into the SEM under ambient conditions in less than five minutes to minimize surface reactions with the atmosphere. Secondary electron images were taken simultaneously with an Everhart-Thornley (SE2)^40^ and an In-lens detector. The former shows a combination of surface topography and material contrast^40^, whereas the latter is only sensitive to the topography, as described in more detail in Section S3 of the Supplementary Materials. To visualize the dynamics of droplet movement, we compiled image sequences to four stop-motion video clips (V1–V4) which are available as Supplementary Video Sequences. Details of the videos can be found in Section S3 of the Supplementary Materials. V1–V3 were recorded slightly above T_EC_ at 250 °C, V4 at 350 °C, with estimated accuracies of ±25 °C in either case. Representative still images from video sequence V1 are compiled in Fig. 4.

The video sequences clearly show that each Sn droplet defines the very location of a transformation process that converts the intact Ge_1−x_Sn_x_ film at its leading edge into liquid Sn and a corrugated trail region. The volume of the Sn precipitates increases gradually as a function of time until the droplets come to a halt as they run into trail regions of other droplets. This can be seen in the sequence of still images in Fig. 4 that are extracted from video sequence V1. The sequence follows the movement of the central one of three large Sn droplets (white arrow in Fig. 4(a)), until it is stopped by the trail of another droplet crossing its path (Fig. 4(b) and (c)). Evidently, the Sn precipitates can only move if they are simultaneously in contact with an intact region of the GeSn layer and the corrugated trail region. From the large trails smaller, oblate droplets start to move into intact regions of the GeSn film. These behave in a similar way as the large droplets, thus carrying the transformation process in an avalanche-like manner into pristine areas between the trails of large dots. Video sequences V2 and V3 in the Supplemental Materials show additional experiments with further details of droplet movement and its final arresting. Video sequence V4, which shows annealing at a higher annealing temperature of T_A_ = 350 °C, demonstrates the remarkable efficiency of the underlying transformation mechanism at a temperature that is still moderate in comparison with typical processing temperatures in standard Si technology.

From the video sequences, we estimated the velocity of the Sn droplets, and thus the speed of the transformation front. We found that the velocity increases with droplet size (for more details see Section S3 of the Supplementary Materials) and reaches values of a few tenth of a micrometer per second both at annealing temperatures of 250 °C and 350 °C. Especially in video sequence V4 one can see the correlation between droplet size and velocity. The higher annealing temperature leads to a more homogeneous transformation front that is defined by droplets of comparable sizes. In contrast, at the lower annealing temperatures employed for V1–V3 only a few large droplets run ahead, whereas the areas between them are gradually filled in by significantly smaller droplets launched from the trails of the forerunners. At the higher temperature of experiment V4 we also observe a more frequent occurrence of trails along the less favorable <100> directions, even though the <110> directions are still preferred.

**Figure 5:**
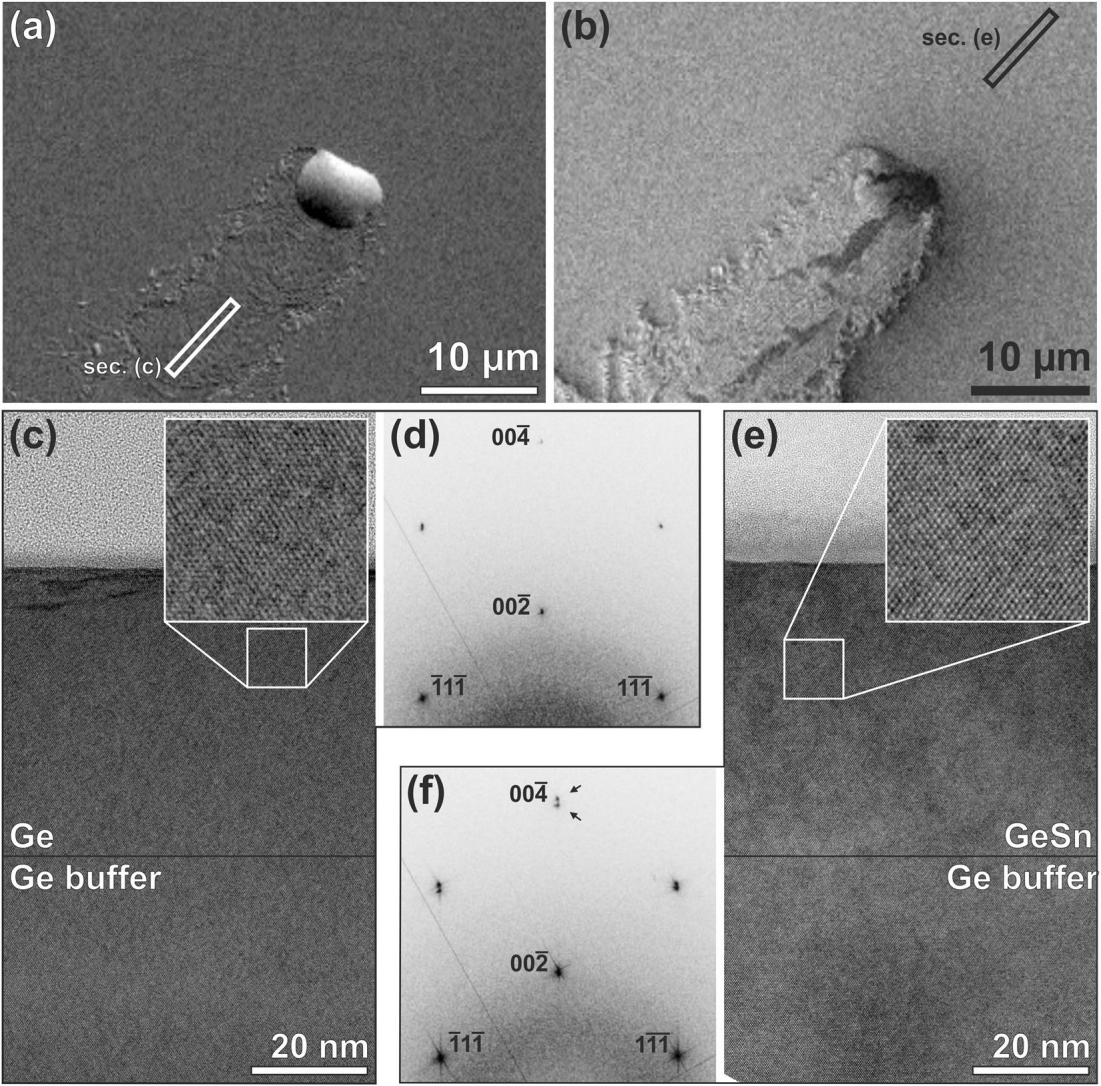
SE2 (**a**) and InLens (**b**) images of a large Sn droplet at the phase separation front after *in-situ* annealing and cool-down. (**c**) displays a HRTEM cross-sectional image from the trail region marked by a rectangle in **(a**). The calculated diffraction pattern (FFT) of (**c**) is displayed in (**d**). (**e**) and (**f)** show the HRTEM cross-sectional image and the FFT diffraction pattern from the strained GeSn region ahead of the Sn droplet that is indicated by a rectangle in (**b**). The two enlarged insets highlight the lattice plane fringes of the HRTEM images. For the FFTs the entire images of (**c**) and (**e**) were used.

**Figure 6:**
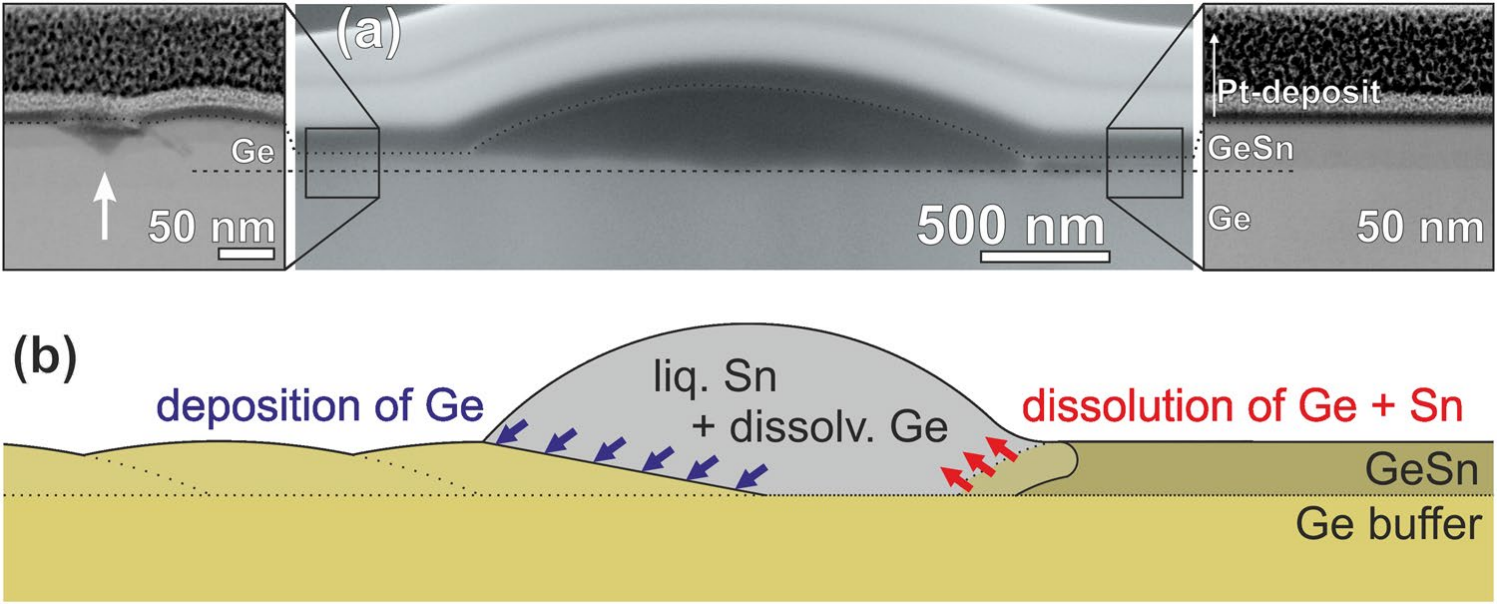
(**a**) Cross-sectional SEM image of a solidified Sn droplet that separates the re-deposited Ge-layer on the left side from the intact GeSn film on the right side. To protect the droplet during FIB-preparation the sample was covered with e-beam- and FIB-induced Pt-deposits that appear as dark- and light-grey conformal layers (indicated in the right inset: Pt-deposit and Ge-Sn are separated by a dotted line). The insets show at the right side the pristine GeSn layer and on the left side the re-deposited Ge film with a small Sn-droplet remaining in a {111} pit (white arrow). The original GeSn-Ge interface is indicated by a dashed line. (**b**) Schematic view of the proposed phase separation process induced by the liquid Sn droplet.

To gain quantitative information on the role of the molten Sn precipitates in the transformation process, we performed TEM experiments on samples from Series D (Table 1) after *in-situ* annealing and subsequent solidification during cool-down. For this purpose, a 20 µm long TEM-lamella was cut with a focused ion-beam (FIB) through a large Sn droplet along its trail and the projected trajectory. A pair of electron transparent windows was then prepared in regions ahead and behind the Sn droplet (marked by rectangles in Fig. 5(a),(b)) to determine the local compositions and strain states in these regions. The high-resolution TEM (HRTEM) images in Fig. 5(c) and (e) reveal excellent crystal quality in either region. Employing EDXS, we found that the trail region consists of almost pure Ge with x ≤ 1%. Only the topmost few monolayers of the film contain segregated Sn. The corresponding lattice constants were extracted by Fourier transformations (FFT) of cross-sectional areas that contain both the Ge substrate and the annealed epilayer. As a result, we identified the trail region to consist of coherent and virtually strain-free Ge, whereas the GeSn film ahead of the Sn droplet preserved the strain and composition of the as-grown epilayer (Fig. 5(d) and (f)). These findings can also explain the observed trenches formation between the strain-free Ge trails and the fully strained GeSn surroundings (line scan L1 in Fig. 3(a)). Evidently, the large mismatch of the respective out of-plane lattice constants renders the formation of an interface between the two regions energetically unfavorable. The TEM images also revealed that the small Sn droplets in the trail (white arrows in Fig. 2(c)) decorate {111}-faceted pits^39^ (left zoom-in in Fig. 6(a)).

In a final experiment, we prepared cross-sectional TEM-lamellae through large Sn droplets, which we found to extend all the way down to the interface with the Ge buffer (Fig. 6(a)). We also used such a specimen to determine the crystal structures and orientations of the different phases after cool-down (Supplementary Materials Section S4).

## Discussion

Our experiments showed that above the eutectic temperature free-running liquid Sn droplets induce the phase separation of a strained GeSn film into liquid Sn and crystalline Ge with negligible Sn content. This process is schematically illustrated in Fig. 6(b). In the overall process, Ge is removed from the leading edge of the droplet, transported through the melt and re-deposited at the trailing edge. It is this directional flow of Ge that causes droplet movement, while simultaneously the dissolved volume of the strained and metastable Si_1−x_Ge_x_ layer becomes converted into unstrained, crystalline Ge and liquid Sn.

This process differs substantially from phase separation of immiscible solids based on solid-state diffusion, such as, the precipitation of Si in the Si/Al system^41^ or topological transitions and surface-mediated growth of nanostructures formed by immiscible phases in the PbTe/CdTe^42^ or the ErSb/GaSb^43^ heterosystems, respectively. Instead, the movement of a Sn droplet resembles more the process of a free-running n-alkane droplet containing surface-active agents^44^. If the latter form grafted hydrophobic layers on an originally hydrophilic surface, droplet movement into hydrophilic areas becomes initiated^44^. In this particular system, contact to hydrophilic and hydrophobic regions can easily be distinguished by the contact angle of the liquid, which is shallower in regions where the droplet wets the surface. A related behavior can be qualitatively observed in our *in-situ* video sequences (Supplementary Materials Section S3) in which the Sn droplets are in the liquid state and moving: Under this condition the contact angle of the Sn droplet is shallower in the GeSn region in front of the droplet, and steeper on the side of the trail, indicating a higher degree of wetting in the former region. A quantitative determination of the contact angles in our system is difficult. Certainly, the contact angles would provide information on the local interface energies, which, however, should depend in a complex way on the exact interface orientation, as well as on the surface reconstructions and local compositions of both the Sn-melt and the Ge_1−x_Sn_x_-layer. Thus, it appears to be more promising to develop a general model for the underlying physics of the observed phase separation process.

A microscopic description of the phase separation process induced by the moving Sn droplets is reminiscent of liquid phase epitaxy (LPE), a technique in which a precursor species is dissolved in a supersaturated melt from where it precipitates epitaxially when brought in contact with a crystalline substrate^45^. In conventional, homoepitaxial LPE, feeding of the melt with the precursor and epitaxial growth are two separate processes that have to be conducted at different temperatures in order to achieve supersaturation in the melt. In our particular case, feeding and growth happen simultaneously in each and every free-running Sn droplet, as long as it stays in contact with both the metastable GeSn layer and the Ge trail.

In analogy to conventional LPE, epitaxial Ge growth at the trailing edge of each droplet is described by a negative difference of the Gibbs free energies of melt and crystalline Ge, ${\rm{\Delta }}{G}^{Ge}$ ^46,47^.

1 $${\rm{\Delta }}{G}^{Ge}=({\mu }_{s}^{Ge}-{\mu }_{l})+{\rm{\Delta }}{\gamma }_{ls}^{Ge} < 0.$$

Here, $({\mu }_{s}^{Ge}-{\mu }_{l})$ is the difference between the chemical potentials of Ge in the solid trail (${\mu }_{s}^{Ge}$) and in the melt (${\mu }_{l}$); ${\rm{\Delta }}{\gamma }_{ls}^{Ge}$ accounts for interface energy differences induced by changes of the liquid-solid interface during growth. The observed low-energy facets in the herringbone pattern of the deposited Ge trails result from a minimization of the ${\rm{\Delta }}{\gamma }_{ls}^{Ge}$ term. Under these conditions LPE growth is essentially defined by $({\mu }_{s}^{Ge}-{\mu }_{l}) < 0$, which describes the aforementioned supersaturation of the melt with Ge^46^.

The feeding part of the LPE process occurs at the leading edge of each Sn droplet, where it is in contact with the strained GeSn film. This contact region is described by ${\rm{\Delta }}{G}^{GeSn}$, which contains additional terms that account for the strained GeSn heterostructure. ${\rm{\Delta }}{G}^{GeSn}$ has to be positive, because here the GeSn film dissolves in the melt. Overall, we get^48^:

2 $${\rm{\Delta }}{G}^{GeSn}=({\mu }_{s}^{GeSn}-{\mu }_{l})+({\rm{\Delta }}{\gamma }_{ls}^{GeSn}+{E}_{ls}+{\gamma }_{ss}) > 0.$$

${\mu }_{s}^{GeSn}$ is the chemical potential of the solid GeSn film; ${E}_{ls}$ accounts for the strain energy of the liquid-solid interface and ${\gamma }_{ss}$ for the solid-solid interface energy between the strained GeSn film and the Ge substrate^48^. Also, ${\rm{\Delta }}{\gamma }_{ls}^{GeSn}\,\,\,$stands for changes in the liquid-solid interface energy. Minimization of this term leads to faceting of the dissolving front, as can be seen nicely in video clip V3 and Fig. S3.3 in Section S3 of the Supplementary Materials.

Feeding and growth are coupled in the droplet by ${\mu }_{l}$, the chemical potential of dissolved Ge in the liquid Sn-melt. If we assume that Ge diffusion in the droplet is much faster than the growth and dissolution kinetics, ${\mu }_{l}$ becomes approximately constant over the melt. ${\mu }_{l}$ is determined by two effects: For one, it depends on the strain- and interface terms in the second pair of parentheses in Equation (2), which becomes a minimum if, as we have observed in the experiments, the whole thickness of the epilayer is dissolved down to the substrate. On the other hand, ${\mu }_{l}$ is determined by the phase separation process itself. Since Ge and Sn are immiscible, their separation into pure Ge and a Sn melt is energetically favorable, releasing essentially the mixing enthalpy ${\rm{\Delta }}{H}_{mix}^{GeSn}$ of the Ge_1−x_Sn_x_ layer. However, producing a Sn melt supersaturated with Ge costs energy. This we express by ${\rm{\Delta }}{G}_{l}^{Ge}$ which is a function of the Ge concentration (and thus of ${\mu }_{l}$) in the melt. This leads then to a change of the Gibbs free energy associated with the phase separation process of:

3 $${\rm{\Delta }}{G}^{Ge+Sn(l)}={\rm{\Delta }}{G}_{l}^{Ge}-{\rm{\Delta }}{H}_{mix}^{GeSn}$$

A more detailed discussion of Equation (3) can be found in the Supplementary Materials, Section S5.

The contributions ${\mu }_{s}^{GeSn}$ and ${\rm{\Delta }}{H}_{mix}^{GeSn}$ in Equations (2) and (3) are only known for equilibrium processes^17,49–51^, but not for our case of far-from-equilibrium MBE-growth. It is, however, clear from the experimental observations that the system gains free energy when Ge and Sn from the metastable GeSn film become dissolved in the Sn melt, and simultaneously almost pure (x ≤ 1%)^17^ Ge is deposited epitaxially at the opposite side of the melt.

To estimate the steady-state Ge concentration in the larger Sn droplets during their movement, we evaluated TEM images after cool-down. During solidification, the Ge-content in the Sn droplet is reduced to the equilibrium solubility at the eutectic point^17^ (Fig. S4.2 in the Supplementary Materials). This effect leads to precipitation of the excess Ge content in the shape of a collar around the Sn-droplet, which can be seen in Fig. 2(b), and in Fig. S4.1 in the Supplementary Materials. An estimate of the collar’s volume with respect to the volume of the solidified Sn core led us to the conclusion that more than 11% Ge must have been dissolved in the liquid droplet. This value is much higher than the equilibrium solubility of 2-3% in the investigated temperature window between T_EC_ and 350 °C (ref.^17^. and Fig. S4.2). Evidently, the dissolution of Sn and Ge from the strained and metastable SnGe film leads to a high degree of supersaturation in the melt, which induces Ge epitaxy at the trailing edge.

### Implications for the Application of GeSn Films

Efficient phase separation at temperatures as low as 230 °C imposes severe limitation to potential applications based on metastable GeSn films. It is therefore a necessary precondition for any conceivable application to suppress Sn precipitation or at least to shift it to higher processing temperatures. Several such measures appear feasible: (i) Capping of the GeSn epilayers affects Sn diffusion in the solid phase and thus delays the formation of sufficiently large Sn precipitates that are required to initiate the transformation process. (ii) Rapid thermal annealing, which is routinely applied in high-temperature CMOS processes, limits the amount of segregated Sn^36^, as compared to the long-term, quasi-equilibrium annealing conditions employed in our experiments. (iii) Growth by CVD under kinetic conditions very far from equilibrium has been shown to suppress Sn precipitation during growth up to temperatures of at least 390 °C^15^. This extension of the metastable range to temperatures substantially above T_EC_ is most likely based on two beneficial properties of CVD. For one, hydrogen termination of the surface during CVD is expected to efficiently suppress surface segregation of Sn, and thus the initial source of molten Sn during growth. Secondly, suppressed Sn segregation and far-from-equilibrium growth conditions allow for higher growth temperatures, thus reducing the density of point defects that may play a role for solid-state Sn diffusion in low-temperature MBE material. Still, the results in ref.^34^ gained from such CVD materials suggest that Sn precipitation can be shifted to higher processing temperatures, but not totally suppressed.

### Summary

In summary, we investigated the thermal stability of uncapped Ge_0.9_Sn_0.1_ films grown by MBE on Ge(001) substrates. Above the eutectic temperature of 231 °C, we find an efficient phase separation mechanism based on molten Sn precipitates that move in a self-propelled manner over the surface. The free-running Sn droplets induce phase separation by taking up Sn and Ge from the pristine GeSn film at their leading edge, and precipitating crystalline Ge at their trailing edge. This behavior is attributed to a liquid-phase epitaxial process that is driven by the free-energy difference between the GeSn and the Ge regions which are both in contact with the molten Sn droplet during movement.

## Methods

### Epitaxial Growth

All samples were grown in a Riber Siva 45 MBE facility with electron-beam evaporators for Si and Ge. For this work, we installed an additional effusion cell filled with high-purity Sn which was calibrated by secondary-ion mass spectrometry (SIMS) of GeSn superlattices in an analogous way as described in ref.^52^. The samples are heated radiatively with temperature control calibrated to within ±25 °C. Polished Ge (001) substrates with a diameter of 100 mm and a specified resistivity of 8 Ωcm were purchased from *Umicore* and subsequently diced into 9.5 × 9.5 mm² pieces to fit into solder-free adapters milled from high-purity Si ingots^53^. Commercial Ge(001) substrates were employed to rule out any influence of the high threading dislocation densities and local strain variations typically associated with virtual (*alias* Ge-buffered) substrates^15,35^. The Ge substrate pieces were chemically pre-cleaned^54^ immediately before being introduced into the load-lock chamber of the MBE system. Before growth, the Ge-substrates were degassed for 30 min at 300 °C and then heated for 15 min to 750 °C for oxide desorption. Growth always commenced with a 50 nm thick Ge buffer layer deposited at 400 °C, which results in smooth surfaces with double-atomic height steps only^53^. The substrate was then ramped down to the respective growth temperature T_G_ of the Ge_1−*x*_Sn_*x*_ epilayer (Table 1).

To assess the thermal stability and the precipitation kinetics of Ge_1−*x*_Sn_*x*_ epilayers under systematic and well-controlled experimental conditions we grew four series (A–D) of un-capped Ge_1−*x*_Sn_*x*_ films (Table 1). Most of the samples of Series A were grown at a low temperature of T_G_ = 120 °C to calibrated our sources and growth parameters, and to assess the composition range x in which we can achieve coherent growth of metastable Ge_1−*x*_Sn_*x*_ without precipitation. In addition, we also grew two samples (A8 and A9 in Table 1) at T_G_ = 200 °C and at a higher growth rate to study the influence of growth temperatures slightly below the eutectic temperature of the Ge/Sn system. All samples of Series A were characterized by X-ray diffraction and atomic force microscopy. Samples A1–A4 and A9 were used for Rutherford Backscattering experiments, and samples A7 – A9 were investigated by transmission electron microscopy.

In Series B to D we concentrated on an application-relevant^15^ Sn concentration of x = 10% and performed different temperature stability experiments. In Series B we increased systematically the growth temperature T_G_, whereas in Series C films grown at T_G_ = 200 °C were *in-situ* annealed in the ultra-high vacuum environment of the MBE chamber for 15 min at increasing temperatures. Finally, we used samples from Series D, which were grown under the same conditions as those of Series C, to perform *in-situ* annealing in the high-vacuum environment of a LEO Supra 35 scanning electron microscope with a heatable sample stage. Transfer from the growth chamber to the SEM occurred under ambient conditions in less than five minutes to minimize surface reactions with the atmosphere. The film compositions, growth rates and film thicknesses of the four investigated sample series are listed in Table 1.

### X-Ray Diffraction (XRD)

All samples were characterized by XRD on either a Seifert XRD 3003 or a PANalytical X’Pert MRD XL diffractometer, both equipped with line detectors. Routinely, rocking curves (ω−2θ scans)^37^ were recorded to determine the out-of-plane lattice constants of the grown GeSn films. We also recorded reciprocal space maps^37^ to assess potential deviations from pseudomorphic film growth.

### Rutherford Backscattering (RBS)

Since XRD can only provide the lattice constants of strained epitaxial films, we also performed RBS experiments on samples A1–A4 and A9 to assess the composition of the respective films. By comparison with the XRD experiments we then determined the relation between composition and lattice constant (Supplementary Materials, Fig. S1.1). The RBS measurements were conducted at the Atomic-Physics and Surface-Science Division at Johannes Kepler University Linz, Austria, in a high vacuum chamber with a base pressure in the 10^−7^ mbar range that is attached to an AN-700 van de Graaf accelerator. The chamber is equipped with two semiconductor surface barrier (SSB) detectors, namely a LN_2_-cooled high-resolution detector^55^ featuring ≈3 keV full-width-at-half-maximum (FWHM) for protons and ≈7 keV for helium ions (scattering angle 150.1°, Cornell geometry), and a standard SSB detector of larger solid angle (scattering angle 154.6°, IBM geometry). Energy spectra of the samples were recorded using 550 keV He^+^ ions. To avoid channeling effects and to optimize depth resolution, two angles of incidence of the ion beam (α = 0° and α = 60°) were chosen. The respective Sn contents were deduced from simulations of the experimental spectra employing the SIMNRA simulation software^56^.

### Scanning Electron Microscopy (SEM)

For investigations of the Sn precipitation kinetics, we constructed a heatable sample holder with built-in temperature sensor to fit into a LEO Supra 35 SEM. This field-emitter SEM is equipped with a Zeiss GEMINI column that allows for in-lens (inLens) detection of so-called SE1 secondary electrons which are predominantly generated by the incident electron beam. In addition, a conventional Everhart-Thornley (SE2) detector is available, which provides a higher degree of material contrast due to a higher sensitivity to SE2 and SE3 electrons that are generated by back-scattered electrons on the sample surface and at structural components of the SEM instrument, respectively^40^.

### Atomic Force Microscopy (AFM)

A Digital Instruments Veeco Dimension 3100 AFM was used in a non-contact tapping mode to assess both the surface roughness and height profiles of the trails left behind by Sn precipitates moving on the surface. Either MICRON or Olympus TESP cantilevers were employed for this purpose. Height images and surface angle plots^57^ were extracted from the AFM raw data with the free Gwyddion analysis software^58^.

### Transmission Electron Microscopy (TEM)

TEM was performed either at the Karlsruhe Institute of Technology (KIT, Karlsruhe, Germany), with a FEI TITAN^3^ 80–300 at 300 kV, or in Linz with a JEOL JEM-2200 FS at 200 kV. Specimens were either prepared by conventional dimple grinding and subsequent argon sputtering in Karlsruhe, or in Linz with the focused ion beam (FIB) technique using a ZEISS 1540XB Cross-Beam system. The FEI TITAN^3^ is equipped with an image aberration corrector, which was used for high-resolution (HR)TEM investigations. Also, scanning TEM (STEM) experiments were performed with the FEI TITAN^3^ in combination with energy dispersive X-ray spectroscopy (EDXS) for composition mappings. The annealed samples were investigated with the JEM-2200 FS using both HRTEM imaging and STEM-EDXS.

## Electronic supplementary material

Supplementary Materials

Supplementary Video Sequence V1

Supplementary Video Sequence V2

Supplementary Video Sequence V3

Supplementary Video Sequence V4
