# Supplementary material for: Free-running Sn precipitates: an efficient phase separation mechanism for metastable Ge1−xSnx epilayers
Source: Sci Rep. 2017 Nov 23;7:16114. doi: 10.1038/s41598-017-16356-8 (PMC5700949; doi:10.1038/s41598-017-16356-8)
Supplement: Supplementary file 1 — Supplementary Materials [file 41598_2017_16356_MOESM1_ESM.pdf]

Supplementary Materials for:

# Free-running Sn precipitates: an efficient phase separation mechanism for metastable $Ge_{1-x}Sn_x$ epilayers

H. Groiss[1, 2, 3, 4, ♣], M. Glaser[1], M. Schatzl[1], M. Brehm[1], D. Gerthsen[4], D. Roth[5], P. Bauer[5], and
F. Schäffler[1]

[1] Institute of Semiconductor and Solid State Physics, Johannes Kepler University Linz, Altenberger Str. 69, 4040 Linz, Austria

[2] Center of Surface and Nanoanalytics (ZONA), Johannes Kepler University Linz, Altenberger Str. 69, 4040 Linz, Austria

[3] CEST Competence Center for Electrochemical Surface Technology, Viktor Kaplan Straße 2, 2700 Wiener Neustadt, Austria

[4] Laboratory for Electron Microscopy, Karlsruhe Institute of Technology, Engesserstr. 7, 76131 Karlsruhe, Germany

[5] Institute of Experimental Physics, Division Atomic Physics and Surface Science, Johannes Kepler University Linz, Altenberger Str. 69, 4040 Linz, Austria

♣ corresponding author, heiko.groiss@jku.at

**Supplementary Material S1 - Experimental Results of Reference Sample Series A**

The reference samples of Series A in Table 1 of the main text were used to calibrate our sources and growth parameters, and to assess the composition range x in which we can achieve in our MBE system coherent growth of metastable $Ge_{1-x}Sn_x$ films on Ge(001) without Sn precipitation. The results are summarized in Fig. S1.1 for 30nm thick $Ge_{1-x}Sn_x$ films in the composition range $4.9\% \leq x \leq 14.5\%$. A low growth temperature of $T_G = 120°C$ was chosen for most samples, but we confirmed that at an increased deposition rate virtually identical results can be achieved at $T_G = 200°C$ (samples A9, A10 in Table 1). Fig. S1.1(a) displays experimental X-ray rocking curves of the out-of-plane 004 reflex together with pendellösung simulations[1] that contain the film thickness and the lattice constant of the respective $Ge_{1-x}Sn_x$ film as the only adjustable parameters. Up to a Sn concentration of 13.6% the rocking curves show very well behaved pendellösung fringes of the thin, compressively strained $Ge_{1-x}Sn_x$ films. Above $x \approx 14\%$ the rocking curve still shows a distinct peak related to strained $Ge_{1-x}Sn_x$, but the fringes are almost completely gone. Reciprocal space maps (Fig. 1(c) in the main text) showed that all epilayers up to 13.6% Sn content were fully strained (pseudomorphic). This finding is confirmed by the absence of dislocations in the cross-sectional TEM images (see Fig. S1.2 (b) below).

To assess the chemical composition of the films, Rutherford backscattering (RBS) experiments were performed on samples A1 – A4 and A9. As a representative example, Fig. S1.1(b) shows the RBS measurement of sample A9 together with the simulation curves for an absolute Sn concentration of $11\pm1\%$. The experiments were repeated under different incidence angles of the 550 keV $He^+$ ions to rule out channeling effects.

From the results of the X-ray rocking curves and a set of five RBS measurements we extracted the relation between the out-of-plane lattice constant and the composition of the film. The experimental data points are plotted in Fig. S1.1(c) together with a least square fit to the experimental data (black dashed line in Fig. S1.1(c)). Also, Vegard's law for the out-of-plane lattice constant of fully strained GeSn films is plotted as a red line in Fig. S1.1(c). For Vegard's law we assumed a linear relation between the out-of-plane lattice constant and composition, utilizing the elastic constants of $\alpha$-Sn that were employed in Ref. 2. The least-square fit is indistinguishable from Vegard's law within experimental accuracy, thus demonstrating its applicability up to x = 14%. This finding is in agreement with the results in Ref. 2, but we did not find any indications for the deviations from Vegard's law above x = 8% that were claimed in Ref. 3. Also, we cannot confirm the rather large bowing parameters predicted by recent density functional simulations[4] which are also depicted in Fig. S1.1(c) as reference lines.

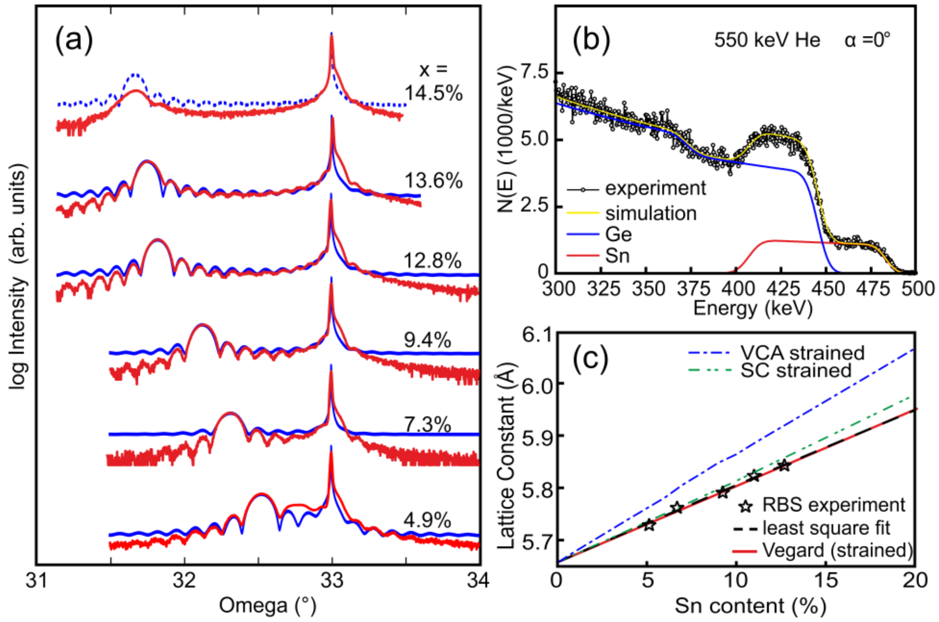


**Fig. S1.1**: (a) X-ray rocking curves of GeSn layers with increasing Sn content. The blue curves represent the respective simulations. (b) Representative RBS experiment on sample A9 (Table 1 in the main text) and corresponding simulation for x = 11±1%. (c) Out-of-plane lattice constant of $Ge_{1-x}Sn_x$ with respect to the Sn content. The red line represents Vegard's law for fully strained, pseudomorphic GeSn on Ge(001), using the elastic constants of $\alpha$-Sn employed in Ref. 2. Vegard's law coincides with the black dashed line that represents a least-square fit to the RBS data points. The dash-dotted curves are taken from two recent theoretical models from Ref. 4.

Based on the results from Series A, the compositions of all subsequently analyzed samples were derived from X-ray diffraction experiments under the assumption of Vegard's law. Sample Series A demonstrates that MBE growth can lead to high-quality $Ge_{1-x}Sn_x$ films up to Sn concentrations of at least 14%, thus confirming earlier findings of other groups[5, 6, 7].

Several samples from the calibration series A were investigated with transmission electron microscopy (TEM) either by scanning TEM (STEM) imaging with bright field (BF) and high-angle annular dark field (HAADF) detectors, or by high-resolution TEM (HRTEM) in parallel illumination mode. The latter mode was applied to cross-sectional specimens for an evaluation of the crystal quality. Local lattice parameters were determined from reciprocal space images generated by fast Fourier transformation (FFT) from HRTEM images. Figures S1.2(a)-(c) present TEM results of sample A5 with x = 0.136, and Figs. S1.2(d)-(f) of sample A7 with x = 0.15. Figures S1.2(a) and (d) show BF images of the two layers. In sample A5 we found a continuous layer of high crystal quality, as can be seen in the corresponding HRTEM image (Fig. S1.2(b)). The sample with 15% Sn contains extended defects (marked with black arrows in Fig. S1.2 (d)), which reach from the surface to the $Ge_{1-x}Sn_x$/Ge interface. The HRTEM image in Fig. S1.2(e) shows that the crystal quality is still high in the surroundings of the defect. The quality of the lattice fringes decreases at defects (marked with a white arrow),

indicating heavy lattice distortion that might be induced by strong relaxation effects or, perhaps, by Sn interstitials. Figures S1.2(c) and (f) show the corresponding FFT reciprocal space images that contain information from both the GeSn layer and the Ge substrate. Only a common *fcc*-based pattern (diamond structure) in $\langle 110 \rangle$ direction is visible, which excludes the presence of precipitates with a different crystal structure. The two zoom-ins (Figs. S1.2(c) and (f)) show the $(006)$ and $(4\bar{4}0)$ reflexes, which are assigned by labels to their corresponding origin in the material stack. The $Ge_{1-x}Sn_x$ layer with x = 13.6% is coherently strained (i.e., tetragonally distorted), which leads to the coincidence of the oblique $(4\bar{4}0)$ spots of the Ge buffer and the GeSn layer in Fig. S1.2(c).

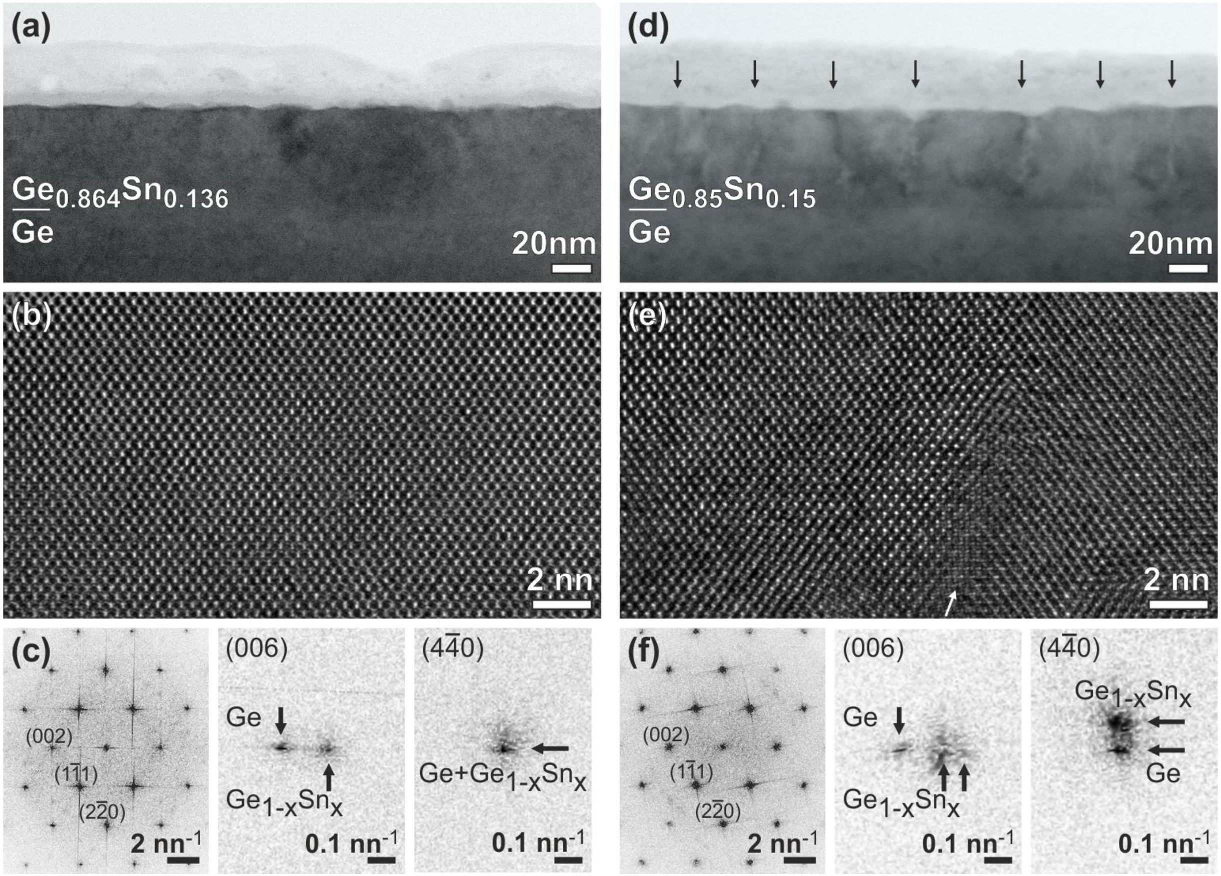


**Fig. S1.2**: (a) and (d) show larger-area BF TEM images of sample A5 (x = 0.136) and A7 (x = 0.15), (b) and (e) the corresponding HRTEM images. With increasing Sn content crystal inhomogeneities and blurred lattice fringes appear (white arrow in (e)). Corresponding reciprocal space images and zoom-ins of the $(006)$ and the $(4\bar{4}0)$ reflexes in (c) and (f) were generated by FFT from HRTEM images that contain both the GeSn film and the Ge substrate.

This is not the case for the GeSn film with 15% Sn. Here, the $(4\bar{4}0)$ in-plane reflex splits (Fig. S1.2(f)), i.e. the film is no longer coherent with the substrate. From the splitting we estimated an average lattice relaxation of 90±10%. Lattice relaxation is not homogeneous, as can be inferred from the fact that the $(006)$ out-of-plane reflex associated with the $Ge_{1-x}Sn_x$ layer

also splits. Thus, under the growth conditions used for Series A, a limit for the substitutional incorporation of Sn into a tetragonally strained $Ge_{1-x}Sn_x$ diamond-lattice is reached between x = 13.6% and x = 15% The incorporation of 15% Sn leads to the observed columnar defects which decrease the layer quality dramatically, with partly relaxed $Ge_{1-x}Sn_x$ defects between high-quality regions. These results are consistent with the loss of pendellösungs fringes in the XRD experiment, which occurs in the same concentration range, as shown in Fig. S1.1.

**Supplementary Material S2 - Temperature Stability during Growth at 300°C**

In sample series B (Table 1 in the main text), we investigated the thermal stability of $Ge_{0.9}Sn_{0.1}$ layers during growth at systematically increasing substrate temperatures. To identify the droplet-like surface features observed in AFM images, we prepared by a focused ion beam (FIB) system a cross-sectional specimen from the sample grown at 300°C (B6) for quantitative TEM analyses. Figure S2(a) displays a cross-sectional scanning electron microscope (SEM) image that was recorded during the preparation of the TEM lamella. From energy dispersive X-ray spectroscopy (EDXS) and HRTEM images, we identified the bright protrusions in the image as β-Sn precipitates. These islands can be associated with solidified Sn droplets that terminate short, braided trails which are visible in the top-view SEM images in Fig. S2(b). The Sn precipitates reach down to the $Ge_{1-x}Sn_x$/Ge interface, as can be seen in cross-sectional TEM image in Fig. S2(c).

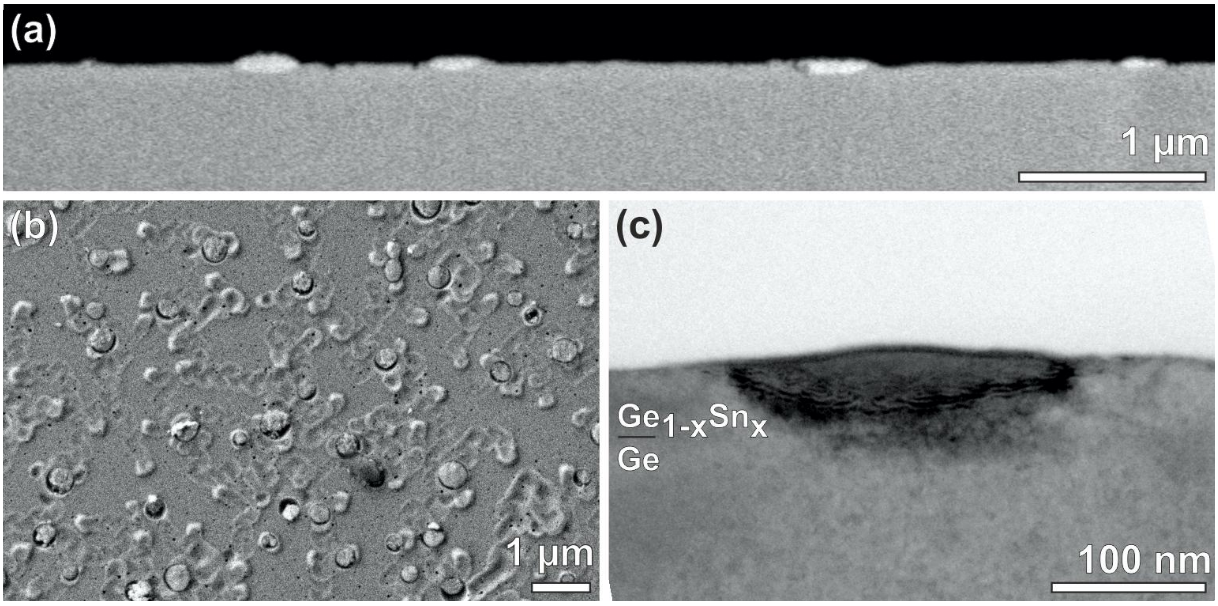


**Fig. S2**: (a) Cross-sectional SEM image of a FIB-cut lamella for TEM investigations. The bright protrusions at the surface are Sn precipitates that can be distinguished from the Ge bulk by material contrast. (b) SEM image of the sample surface where Sn droplets decorate the end of braided trails. (c) Cross-sectional TEM image of a β-Sn precipitate.

**Supplementary Material S3 - Post-Growth SEM-annealing: Stop-Motion Video Sequences**

Stop-motion video sequences of our *in-situ* SEM-annealing experiments are available as video files. Sequences V1 - V3 were recorded slightly above the eutectic temperature $T_E$ at 250±25°C, V4 at 350±25°C. Video sequences V1 - V3 were simultaneously recorded with an Everhart-Thornley detector (labeled SE2 in the movies) and a through-the-lens detector[8] (labeled inLens in the movies). Video sequence V4 is only available in the inLens configuration.

The inLens SEM images show high-resolution topography contrast because mainly the secondary electrons generated directly by the incident electron beam are detected. Topography resolution is worse in the SE2 SEM images because contributions of secondary electrons induced by back-scattered electrons contribute to the detected electron intensity, leading to a blurring of small topography features. Bright regions in SE2 SEM images are observed (e.g. at droplets), if the surface is inclined toward the Everhart-Thornley detector. An interesting feature is found in inLens SEM images (Figs. S3.2 and S3.3 below) where dark regions can be recognized on the Sn droplet as well as in the trail behind the droplets. We associate these intensity changes with a locally higher work function which lowers the intensity of the emitted secondary electrons. Work-function changes can be, e.g., associated with compositional changes in the top few atom layers close to the surface.

The following files are available:

**Video sequence V1; start position depicted in Fig. S3.1:** At the beginning, the video sequence shows three large Sn droplets moving downward on parallel <110> trajectories. Two of them come to a halt when encountering areas that have already been converted from the original GeSn film into re-deposited Ge by secondary droplets launched from the trails of other droplets. The smaller trails of the secondary droplets lead to an avalanche-like broadening of the converted area in the wake of the main trajectory. The trails are well resolved in the inLens video sequence, whereas the corresponding Sn droplets at the trail's ends can only be well recognized in the SE2 sequence. The center one of the three original droplets runs furthest, but it is finally stopped by the trail of a fourth droplet that crosses its trajectory under an angle of 45°. Toward the end of the sequence, this fourth droplet turns counter-clockwise in order to avoid other trails, but finally comes to a halt when it is completely surrounded by re-deposited Ge. This situation is also depicted in the extracted still images that are depicted in Figs. 4(b) and 4(e) in the main text. Video V1 covers a time span of 27 min and 46 s and contains 79 frames, i.e. the average time between frames is ~21 s. From the stop-motion video sequence we determined the velocity of the droplets,

which we found to increase with droplet volume. The smallest droplet (the left one in the initial configuration depicted in Fig. S3.1) starts with a velocity of ≈0.13 µm/s. The other two have velocities of ≈0.16 µm/s. At the end of the video sequence, the two crossing droplets have a speed of ≈0.21 µm/s.

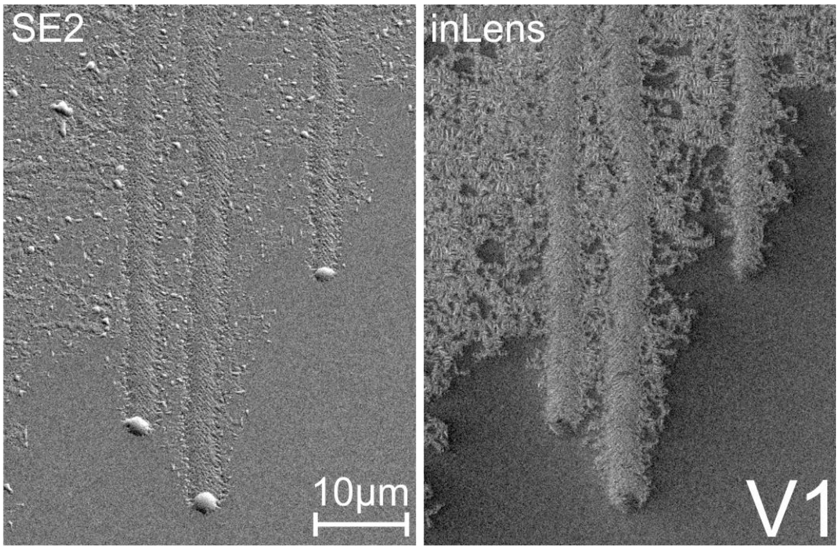


**Fig. S3.1:** Start position of video V1, which was recorded at 250°C.

**Video sequence V2; start position depicted in Fig. S3.2:** A Sn droplet moves until it reaches phase separated material. The video shows a time span of 11 min and 4 s and contains 33 frames (time between frames ~20 s). The average velocity of the droplet is ≈0.21 µm/s.

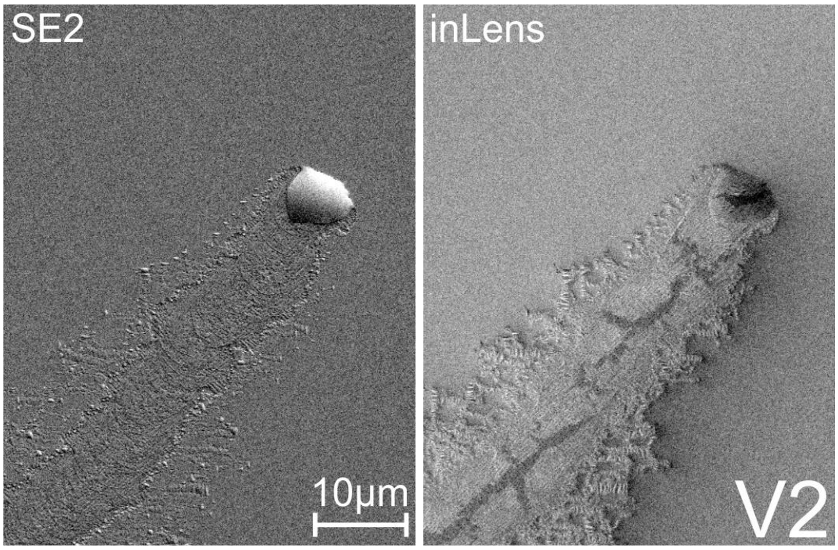


**Fig. S3.2:** Start position of video V2, which was recorded at 250°C. Note the dark regions in the inLens image discussed in the text.

**Video sequence V3; start position depicted in Fig. S3.3:** A large Sn droplet is moving in a <100> direction in close vicinity to already transformed material until it finally comes to a halt. Note that in this less frequently observed direction of movement the leading edge of the droplet is completely facetted in a saw tooth pattern to minimize the interface energy. The video sequence shows a time span of 9 min and 1 s and contains 24 frames (time between frames ~26 s). The average velocity of the facetted droplet is ≈0.08 μm/s, i.e. significantly slower than droplet movement in the predominant <110> directions.

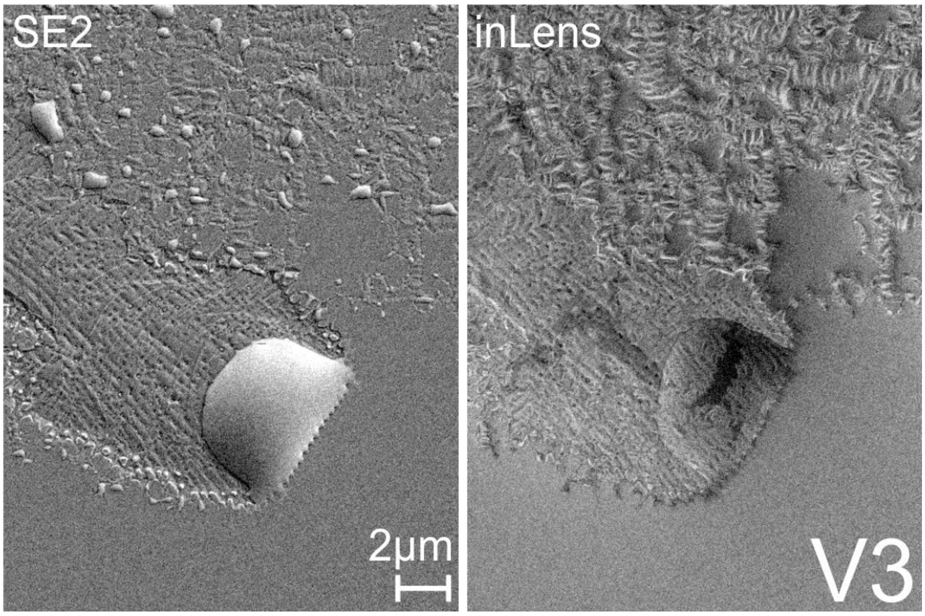


**Fig. S3.3:** Start position of video V3, which was recorded at 250°C. Note the saw-tooth-like faceting at the leading edge of the droplet that moves in this video sequence in an energetically less favorable <100> direction.

**Video sequence V4; start position depicted in Fig. S3.4:** This inLens video sequence was recorded at a somewhat higher annealing temperature of 350±25°C. It shows an overview of the transformation front between the smooth GeSn film in the lower left part of the field of view, and the corrugated area of re-deposited Ge left behind by a large number of moving Sn droplets. Only the trails are resolved here, whereas the droplets at their ends are hardly visible with the inLens detector. This video sequence clearly shows that the avalanche-like cascade of droplets converts essentially the whole GeSn film into a corrugated, single crystalline Ge layer. Only small patches in the corrugated area remain seemingly unaffected. One can, however, observe that some of these decompose by the delayed formation of additional secondary droplets after the main transformation front has passed. Video sequence V4 provides particularly impressive evidence for the efficiency of the phase separation process induced by free-running Sn precipitates at a still moderate temperature.

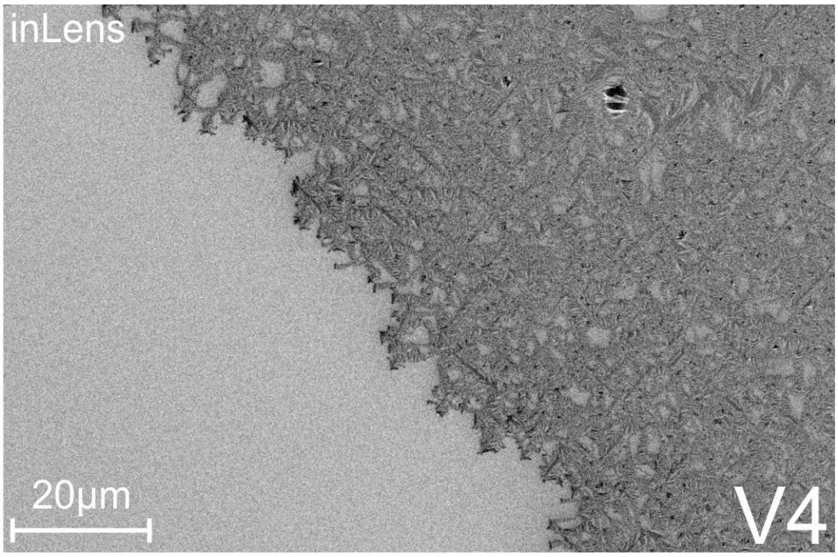


**Fig. S3.4:** Start position of video V4, which was recorded at 350°C.

It is worth to mention that the droplet sizes at the decomposition front increase with time, concomitant with an increase of the average droplet velocity. At the beginning, the droplets move with ≈0.03 µm/s, but reach a velocity of ≈0.15 µm/s at the end of the video. The velocity of the Sn-droplets is essentially defined by the flux of Ge through the Sn melt. The Ge flux is limited by the contact area of the Sn droplet with the GeSn layer, which grows linearly with the droplet radius. The droplet volume, however, which defines the maximum possible Ge content in the melt, grows to first approximation with the third power of the radius. Thus, the flux through the contact area, and therefore the droplet velocity, increases with droplet size. Since Sn from the GeSn layer is collected by the liquid droplets, the droplet size increases with annealing time, and thus also their velocity. Moreover, the droplet velocity should increase with temperature, because diffusion increases with temperature. However, the latter effect is expected to be small in the limited temperature windows used in the experiments, which means that the size effect should dominate the droplet velocity. Video clip V4 covers a time span of 14 min and 43 s and contains 37 frames (time between frames ~24 s).

**Supplementary Material S4 – Precipitate Orientation after Cool-Down**

We prepared a FIB lamella through a large Sn dot of an annealed sample from Series D (Table 1 in the main text) after cool-down to identify the crystal structures and orientation relations of the different phases in the solid state. The droplet reaches down to the original interface between Ge buffer and GeSn film. It crystallizes in the β-Sn crystal structure, as determined by HRTEM imaging. Due to the temperature behavior and the melting temperature of Sn, it is clear that the droplets are liquid during annealing above 230°C. After cool-down, the solidified Sn droplet is surrounded by a collar of roughly triangular cross section (black arrow in Fig. S4.1(a) and white arrow in Fig. 2(b) in the main text), which we identified by EDXS as consisting of pure Ge. This Ge collar forms during cool-down at the liquid-solid phase transition by precipitation of essentially the entire amount of Ge that was originally dissolved in the liquid Sn melt.

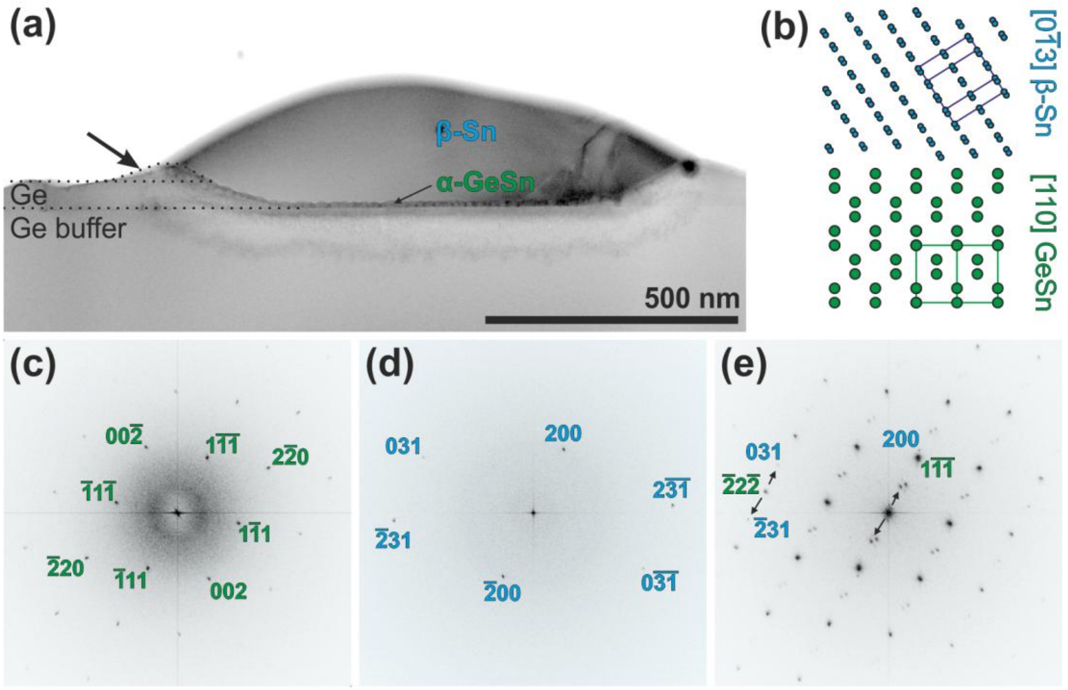


**Fig S4.1**: (a) Cross sectional TEM image of a large solidified Sn droplet which penetrates the whole thickness of the GeSn film down to the Ge buffer. The dotted lines indicate the original Ge substrate and GeSn layer surfaces; the arrow marks the Ge-collar formed during cool-down. (b) Crystal orientation of the β-Sn droplet in relation to the Ge substrate. Reciprocal space images in (c) – (e) were calculated by FFT from HRTEM images covering (c) the Ge buffer region, (d) the β-Sn droplet and (e) the interface region containing the Ge buffer, the droplet and a layer of α-GeSn that crystallizes at the interface. The diffraction peaks are labeled in green for Ge-related signals, and in blue for Sn-related signals.

Such a behavior is consistent with the binary phase diagram of Ge-Sn (Fig. S4.2), which has a eutectic point very close to pure Sn at a temperature about 1°C below the melting point of β-Sn.[9] At temperatures above the eutectic temperature, liquid Sn can take up significant amounts of Ge which have to precipitate as almost pure Ge when the temperature reaches the eutectic temperature. An estimate of the Ge content in the melt based on the relative volume of the Ge collar is given in the main text.

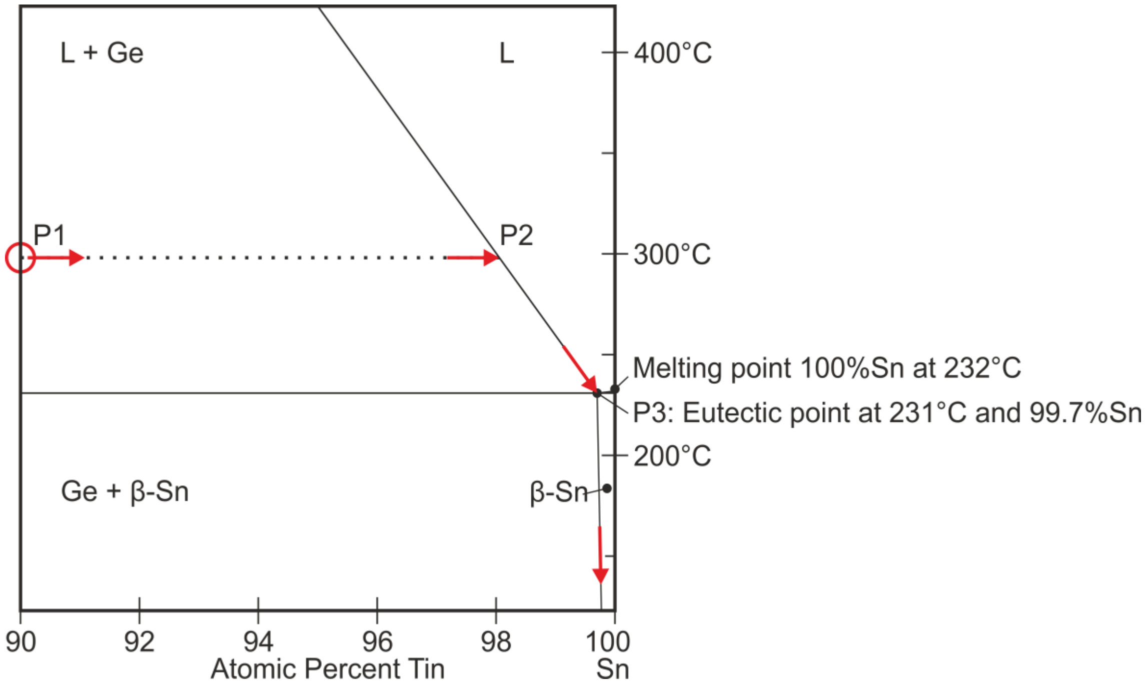


**Fig. S4.2:** Simplified Ge-Sn phase diagram in the relevant, Sn-rich range. The figure is based on the experimental data in Ref. 9.

Fig. S4.2 displays schematically the formation process of the Ge collar surrounding the Sn droplet in the Sn-rich region of the Ge-Sn phase diagram. As discussed in the main text, a supersaturated Ge-Sn solution forms in the moving Sn droplets. As an example, P1 marks a location in the liquid + solid region of the phase diagram that corresponds to a typical experimental condition at 300°C with a supersaturated concentration of 10% Ge. Because in this region liquid Sn co-exists with almost pure, solid Ge, Ge can precipitate at the trailing edge of the droplet, where it forms the observed, faceted Ge trail. As long as the droplet moves, it is fed with Ge from the strained and metastable GeSn layer, and thus stays in the supersaturated region. If the melt is completely surrounded by Ge everywhere, feeding stops and Ge can precipitating around the droplet circumference until point P2 is reached on the equilibrium solidus/liquidus curve in Fig. 4.2. If the Sn-droplet is then cooled down, further Ge will precipitate as the system moves down on the equilibrium curve to the eutectic point

(process path along markings with red arrows). At the eutectic point, the droplet crystallizes as β-Sn with the equilibrium concentration of ≈ 0.3% Ge.

To determine the alignment between the diamond lattice of the Ge film and the tetragonal lattice of the solidified β-Sn droplet, we recorded HRTEM images from different regions of Fig. S4.1(a) along the $[110]$ zone axis of the Ge substrate. Conversions by FFT into reciprocal space images are displayed in Figs. S4.1(c) - (e). The assignment of the diffraction spots refers to the respective crystal structure, with blue labeling being used for β-Sn and green one for Ge. The solidified droplet itself has the β-Sn structure exhibiting the $[0\bar{1}3]$ zone axis. The β-Sn crystal lattice is oriented such that its $(200)$ planes are almost parallel to the $(1\bar{1}\bar{1})$ planes of the diamond lattice of Ge. A schematic representation of the relative alignment of the two lattices is depicted in Fig. S4.1(b). Alignment is not perfect, as can be seen in Fig. S4.1(e), were the $200_{Sn}$ (blue) and the $1\bar{1}\bar{1}_{Ge}$ (green) spots do not perfectly coincide.

Fig. S4.1(a) also shows a thin α–GeSn layer that decorate the interface between the separated Sn and Ge phases. We assume that this layer has formed in the initial stages of crystallization of the Sn droplet. Similar α–GeSn structures can be found e.g. in capped GeSn-layers after annealing[10]. Also, the β-Sn droplets have developed low-energy facets during solidification, which can be seen in the SEM image of Fig 2(b) in the main text and in the asymmetric polygon-contour of the β-Sn region in Fig. S4.1(a).

## Supplementary Material S5 –Decomposition Thermodynamics[11]

One can write the Gibbs free energy $G^{GeSn}$ of $Ge_{1-x}Sn_x$ as the weighted sum of the Gibbs free energies $G^{Ge}$ for a Ge fraction $(1-x)$ and $G^{\alpha-Sn}$ for a $\alpha$-Sn fraction $x$ as

$$G^{GeSn} = (1-x)G^{Ge} + xG^{\alpha-Sn} + \Delta G_{mix}^{GeSn}(x), \tag{S1}$$

with the mixing term

$$\Delta G_{mix}^{GeSn}(x) = \Delta H_{mix}^{GeSn}(x) - T\Delta S_{mix}^{GeSn}(x). \tag{S2}$$

The entropy of formation $\Delta S_{mix}^{GeSn}$ lowers the energy for a solution of two components and often leads to an ideal solution with no miscibility gap. $Ge_{1-x}Sn_x$, however, exhibits a large miscibility gap. Thus, over a large composition range the heat of formation $\Delta H_{mix}^{GeSn}$ must be much larger than $T\Delta S_{mix}^{GeSn}$. Therefore $T\Delta S_{mix}^{GeSn}$ can be neglected over almost the entire composition range, resulting in

$$\Delta G_{mix}^{GeSn} \sim \Delta H_{mix}^{GeSn}. \tag{S3}$$

After decomposition the material is separated into almost pure, re-deposited Ge with a Gibbs free energy $G^{Ge}$ and liquid Sn with a concentration $y$ of solved Ge. The total Gibbs energy of the constituents can be written as:

$$G^{Ge+Sn(l)} = (1-x-y)G^{Ge} + xG^{Sn(l)} + yG^{Ge(l)} + \Delta G_{mix}^{Sn(l)Ge(l)} + \gamma \tag{S4}$$

The first term is for the re-deposited Ge (the < 1% of Sn solved in the crystalline Ge phase is neglected). The second, third and fourth terms describe liquid Sn ($xG^{Sn(l)}$) containing solved Ge ($yG^{Ge(l)}$) and the mixing term ($\Delta G_{mix}^{Sn(l)Ge(l)}$). $\gamma$ is the energy of a general interface between crystalline Ge and liquid Sn. This term will be neglected in the following, because the interface terms are contained in our liquid-phase-epitaxy model (Equs. (1) and (3) in the main text). The energy gain associated with the phase separation into Ge and liquid Sn is the difference between Equ. (S4) and (S1):

$$\Delta G^{Ge+Sn(l)} = G^{Ge+Sn(l)} - G^{GeSn} = y\left(G^{Ge(l)} - G^{Ge}\right) + \Delta G_{mix}^{Sn(l)Ge(l)} + x\left(G^{Sn(l)} - G^{\alpha-Sn}\right) - \\ -\Delta G_{mix}^{GeSn} = \Delta G_l^{Ge} + \Delta G_l^{Sn} \tag{S5}$$

The fraction of solved Ge in liquid Sn is determined by a trade-of between dissolving Ge in the liquid Sn

$$\Delta G_l^{Ge} = y\left(G^{Ge(l)} - G^{Ge}\right) + \Delta G_{mix}^{Sn(l)Ge(l)} > 0 \tag{S6}$$

and forming liquid Sn from a solid α-Sn component releasing the mixing enthalpy $\Delta H_{mix}^{GeSn}$:

$$\Delta G_l^{Sn} = x\left(G^{Sn(l)} - G^{\alpha-Sn}\right) - \Delta H_{mix}^{GeSn} < 0 \tag{S7}$$

$G^{Ge(l)} - G^{Ge}$ is positive, because we are far below the melting point of Ge. We have a supersaturated solution, thus $\Delta G_{mix}^{Sn(l)Ge(l)}$ is also positive, resulting in $\Delta G_l^{Ge} > 0$. Because we are near the melting point of Sn the term $x\left(G^{Sn(l)} - G^{\alpha-Sn}\right)$ should be mainly governed by the enthalpy of fusion $H_{\text{fus}}^{Sn}$ , which is in the order of $xH_{\text{fus}}^{Sn} \approx 0.7\ \frac{\text{kJ}}{\text{mol}}$ for $x = 0.1$.

The mixing enthalpy $\Delta H_{mix}^{GeSn}$ can be dominated by two main terms: the difference in bond energies between Ge-Ge, Sn-Sn and Ge-Sn bonds and the deformation energy induced by atomic size or bond-length mismatch of the constituents. Several publications[12, 10] indicate that the bond energies differences are small. $Sn_{1-y}Ge_y$ melts, for instance, have a minimum in the surface tension, and it is possible to fabricate regular $Ge_{0.5}Sn_{0.5}$ alloys. Both results lead to the conclusion that Ge-Sn bonds are energetically not unfavorable enough to cause the large miscibility gap. It is therefore more likely that the deformation of the Ge-matrix by the much larger Sn-atoms is the dominating term leading to the large $\Delta H_{mix}^{GeSn}$. Our system is grown far away from equilibrium. Therefore, the inner energy, and thus $\Delta H_{mix}^{GeSn}$,is also increased by e.g. defects, unsaturated bonds, Sn or Ge interstitials, etc.. We therefore assume $\Delta H_{mix}^{GeSn} \gg x\left(G^{Sn(l)} - G^{\alpha-Sn}\right)$. Thus, we can write for Equ. (S7) $\Delta G_l^{Sn} \sim -\Delta H_{mix}^{GeSn}$ which leads to Equ. (3) in the main text:

$$\Delta G^{Ge+Sn(l)} = \Delta G_l^{Ge} - \Delta H_{mix}^{GeSn} \tag{S8}$$

## References

[1] Pietsch, U., Holý, V., Baumbach, T. *High-Resolution X-ray Scattering*, 2nd ed. (Springer, 2004).

[2] Bhargava, N., Coppinger, M., Prakash Gupta, J., Wielunski, L. & Kolodzey, J. Lattice constant and substitutional composition of GeSn alloys grown by molecular beam epitaxy. *Appl. Phys. Lett.* **103,** 041908 (2013).

[3] Oehme, M. *et al.* Epitaxial growth of highly compressively strained GeSn alloys up to 12.5% Sn. *J. Cryst. Growth* **384,** 71–76 (2013).

[4] Eckhardt, C., Hummer, K. & Kresse, G. Indirect-to-direct gap transitions in strained and un-strained $Sn_xGe_{1 x}$ alloys. *Phys. Rev. B* **89**, 165201 (2014).

[5] Gurdal, O. *et al.* Low-temperature growth and critical epitaxial thicknesses of fully strained metastable $Ge_{1-x}Sn_x$ ($x \lesssim 0.26$) alloys on Ge(001)2×1. *J. Appl. Phys.* **83,** 162–170 (1998).

[6] Deng, X., Yang, B.-K., Hackney, S. A., Krishnamurthy, M. & Williams, D. R. M. Formation of Self-Assembled Quantum Wires during Epitaxial Growth of Strained GeSn Alloys on Ge(100): Trench Excavation by Migrating Sn Islands. *Phys. Rev. Lett.* **80,** 1022–1025 (1998).

[7] Wang, W., Li, L., Tok, E. S. & Yeo, Y. C. Self-assembly of tin wires via phase transformation of heteroepitaxial germanium-tin on germanium substrate. J. Appl. Phys. 117, 0–8 (2015)

[8] Goldstein, J. I. Scanning electron microscopy and X-ray microanalysis, 2nd ed. (Plenum Press, 1992).

[9] Predel, B. Ge-Sn (Germanium-Tin) in *Ga-Gd – Hf-Zr*, *Landolt-Börnstein - Group IV Physical Chemistry* (ed O. Madelung) (Springer, 2013).

[10] Tonkikh, A. A. *et al.* Cubic phase Sn-rich GeSn nanocrystals in a Ge matrix. *Cryst. Growth Des.* **14,** 1617–1622 (2014).

[11] Porter, D. A., Easterling, K. E., Sherif, M. Y. *Phase Transformation in Metals and Alloys*, 3nd ed. (CRC Press, 1992).

[12] Naidich, Y. V. & Perevertailo, V.M. Surface and contact properties in the system composed of germanium and a tin-germanium melt, *Soviet Powder Metallurgy and Metal ceramics* **10**, 142-147 (1971).
